# Supplementary material for: Health-related quality of life of patients with type 2 diabetes mellitus at a tertiary care hospital in Ethiopia
Source: PLoS One. 2022 Feb 18;17(2):e0264199. doi: 10.1371/journal.pone.0264199 (PMC8856533; doi:10.1371/journal.pone.0264199)
Supplement: S1 File — (DOCX) [file pone.0264199.s002.docx]

**Supplementary file 1: An English and Amharic version of EQ-5D-5L**

Under each heading, please tick the ONE box that best describes your health TODAY.

**MOBILITY**

I have no problems in walking about 

I have slight problems in walking about 

I have moderate problems in walking about 

I have severe problems in walking about 

I am unable to walk about 

**SELF-CARE**

I have no problems washing or dressing myself 

I have slight problems washing or dressing myself 

I have moderate problems washing or dressing myself 

I have severe problems washing or dressing myself 

I am unable to wash or dress myself 

**USUAL ACTIVITIES** (e.g. work, study, housework, family or leisure activities)

I have no problems doing my usual activities 

I have slight problems doing my usual activities 

I have moderate problems doing my usual activities 

I have severe problems doing my usual activities 

I am unable to do my usual activities 

**PAIN / DISCOMFORT**

I have no pain or discomfort 

I have slight pain or discomfort 

I have moderate pain or discomfort 

I have severe pain or discomfort 

I have extreme pain or discomfort 

**ANXIETY / DEPRESSION**

I am not anxious or depressed 

I am slightly anxious or depressed 

I am moderately anxious or depressed 

I am severely anxious or depressed 

I am extremely anxious or depressed 


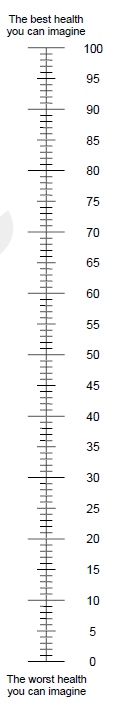
**Part- II**


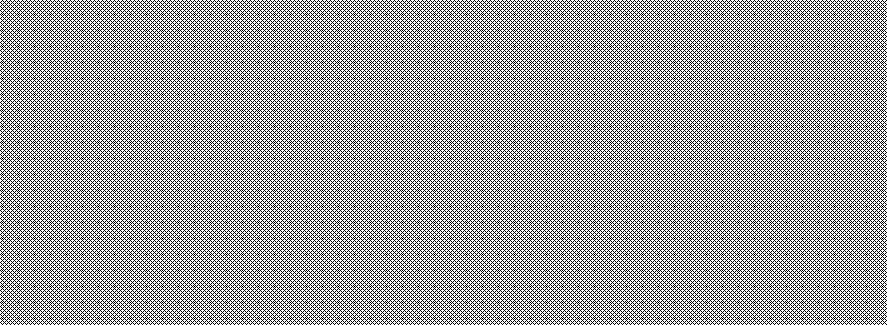


The best health you can imagine

100

95

90

85

80

75

70

65

60

55

50

45

40

35

3

25

20

15

10

5

0

• We would like to know how good or bad your health is TODAY.

• This scale is numbered from 0 to 100.

• 100 answer the best health you can imagine.

• 0 means the worst health you can imagine.

• Mark an X on the scale to indicate how your health is TODAY.

• Now, please write the number you marked on the scale in the box below

YOUR HEALTH TODAY =

**የአማርኛ ትርጉም**

ለኢትዮጵያ በእያንዳንዱ ርዕስ ስር ፣እባክዎ ዛሬ ያለዎትን ጤንነት በተሻለ ሁኔታ የሚገልጸው አንድ ሳጥን ላይ ምልክት ያድርጉ፡፡

**እንቅስቃሴ**

የመራመድ ችግር የለብኝም ❑

አነስተኛ የሆነ የመራመድ ችግር አለብኝ ❑

መጠነኛ የሆነ የመራመድ ችግር አለብኝ ❑

ከባድ የሆነ የመራመድ ችግር አለብኝ ❑

ምንም መራመድ አልችልም ❑ **ራስን መንከባከብ**

ለመታጠብም ሆነ ለመልበስ ምንም ችግር የለብኝም ❑

ለመታጠብም ሆነ ለመልበስ አነስተኛ የሆነ ችግር አለብኝ ❑

ለመታጠብም ሆነ ለመልበስ መጠነኛ ችግር አለብኝ ❑

ለመታጠብም ሆነ ለመልበስ ከፍተኛ የሆነ ችግር አለብኝ ❑

ራሴ ልታጠብም ሆነ ልለብስ አልችልም ❑

**መደበኛ ተግባራት** (ለምሳሌ፦ስራ፣ትምህርት፣የቤት ውስጥ ስራ፣ቤተሰባዊ ወይ ምየእረፍትጊዜተግባራት)

መደበኛ ተግባራቶቼን ያለ ምንም ችግር አከናውናለሁ ❑

መደበኛ ተግባራቶቼን ለማከናወን አነስተኛ ችግር አለብኝ ❑

መደበኛ ተግባራቶቼን ለማከናወን መጠነኛ ችግር አለብኝ ❑

መደበኛ ተግባራቶቼን ለማከናወን ከፍተኛ ችግር አለብኝ ❑

መደበኛተግባራቶቼን ለማከናወን አልችልም ❑

**የሕመም ስሜት/ምቾት ማጣት**

የሕመም ስሜትም ሆነ የምቾት ማጣት ስሜት የለኝም ❑

አነስተኛ የሕመም ስሜት ወይም የምቾት ማጣት ስሜት አለኝ ❑

መጠነኛ የሕመም ስሜት ወይም የምቾትማጣት ስሜት አለኝ ❑

ከባድ የሕመም ስሜት ወይም የምቾት ማጣት ስሜት አለኝ ❑

የከፋ የሕመም ስሜት ወይም የምቾት ማጣት ስሜት አለኝ ❑

**ጭንቀት/ድብርት**

ጭንቀትም ሆነ ድብርት የለብኝም ❑

አነስተኛ ጭንቀት ወይም ድብርት አለብኝ ❑

መጠነኛ ጭንቀት ወይም ድብርት አለብኝ ❑

ከባድ ጭንቀት ወይም ድብርት አለብኝ ❑

እጅግ ከባድ ጭንቀት ወይም ድብርት አለብኝ ❑

**ክፍል II**


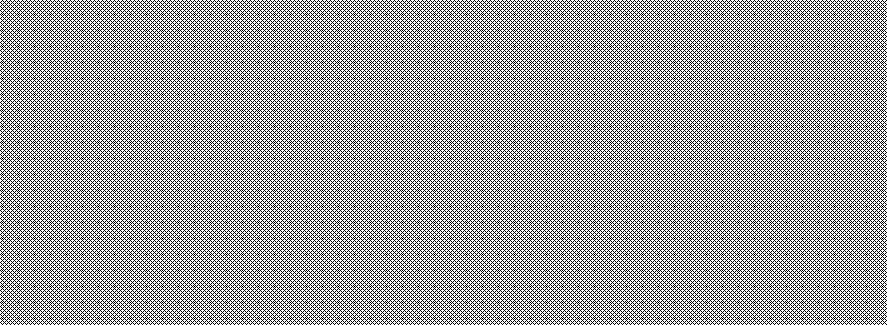


ሊኖርዎ ሚችለው በጣም ጥሩው የጤና ሁኔታ 100

95

90

85

80

75

70

65

60

55

50

45

40

35

30

25

20

15

10

5

በጣም መጥፎ የጤና ሁኔታ

- ዛሬ የጤናዎ ሁኔታ ምን ያህል ጥሩ ወይም መጥፎ መሆኑን ለማወቅ እንፈልጋለን::
- ይህ መለኪያ ከ 0 እስከ 100 ድረስ ቁጥሮች አ ሉት::
- 100 ማለት እርስዎ ሊኖርዎ የሚችለው በጣም ጥሩ የጤና ሁኔታ ነው::
- 0 ማለት እርስዎ ሊኖርዎ የሚችለው በጣም መጥፎ የጤና ሁኔታ ነው ::
- በመለኪያው ላይ ዛሬ ጤንነትዎ ያለበትን ሁኔታ ለማሳየት የ X ምልክት ያድርጉ::
- አሁን እባክዎን ከታች ባለው ሳጥን ውስጥ በመለኪያው ላይ ምልክት ያደረጉበትን ቁጥር ይፃፉ::

የዛሬ ጤንነትዎ =
