# Supplementary material for: Health-related quality of life of patients with type 2 diabetes mellitus at a tertiary care hospital in Ethiopia
Source: PLoS One. 2022 Feb 18;17(2):e0264199. doi: 10.1371/journal.pone.0264199 (PMC8856533; doi:10.1371/journal.pone.0264199)
Supplement: S1 Table — (DOCX) [file pone.0264199.s003.docx]

**S1 Table:** Percentage of self-reported health problems among patients with T2DM using EQ-5D-5L descriptive system

| Variables | | % Reported problems | | | | |
| --- | --- | --- | --- | --- | --- | --- |
|  |  | **Mobility** | **Self-care** | **Usual activities** | **Pain/**  **discomfort** | **Anxiety/**  **Depression** |
| Participants | | **60.5** | **37.2** | **34.1** | **67.3** | **43.8** |
| Gender | | | | | | |
|  | Male | 23.9* | 15.3 | 13.4 | 24.7* | 21.9 |
|  | Female | 36.6 | 21.9 | 20.7 | 42.6 | 21.9 |
| Age category | | | | | | |
|  | < 45 | 2.0 | 0.60 | 0.60 | 2.60 | 2.60 |
|  | 45-64 | 21.3 | 7.10 | 10.8 | 24.1 | 14.8 |
|  | > 65 | 37.2 | 29.5* | 22.7* | 40.6 | 26.4 |
| Marital status | | | | | | |
|  | Married | 43.2 | 21.9* | 22.7 | 47.7 | 34.9* |
|  | Unmarried | 17.3 | 15.3 | 11.4 | 19.6 | 8.90 |
| Educational status | | | | | | |
|  | Illiterate | 20.8 | 17.4 | 15.4 | 25.4 | 9.70 |
|  | Primary school | 17.1 | 10.3 | 8.30 | 19.7 | 12.3 |
|  | Secondary school and higher | 22.8 | 9.7 | 10.5 | 22.5 | 21.9 |
| Occupational status | | | | | | |
|  | Employed | 21.6 | 9.70* | 10.8* | 22.4* | 16.2 |
|  | Non-employed | 38.9 | 27.6 | 23.3 | 44.9 | 27.6 |
| Average household monthly income | | | | | | |
|  | < 14.84$ | 19.7* | 12.0 | 10.5 | 21.9* | 12.3 |
|  | > 14.84 $ | 40.7 | 25.1 | 23.4 | 45.3 | 31.3 |
| Since DM diagnosis | | | | | | |
|  | < 5 years | 12.5 | 4.0* | 4.0* | 12.3 | 10.5* |
|  | 5-10 years | 17.7 | 11.1 | 11.4 | 19.4 | 15.4 |
|  | > 10 years | 30.5 | 22.2 | 18.8 | 35.6 | 17.7 |
| Lifestyle modification used | | | | | | |
|  | Yes | 54.0* | 34.9 | 31.5 | 62.5 | 40.3 |
|  | No | 6.50 | 3.30 | 2.30 | 4.50 | 3.10 |
| Types of lifestyle modification | | | | | | |
|  | Dietary | 40.5 | 25.9 | 22.9 | 46.6 | 29.0 |
|  | Physical activity | 12.2 | 7.60 | 7.90 | 13.7 | 12.5 |
|  | Dietary and physical | 4.90 | 2.10 | 2.10 | 5.80 | 2.40 |
| Comorbidities | | | | | | |
|  | Yes | 48.3* | 30.7* | 27.6* | 55.4* | 33.5 |
|  | No | 12.2 | 6.50 | 6.50 | 11.9 | 10.2 |
| Types of comorbidities | | | | | | |
|  | Hypertension | 31.6* | 19.1* | 14.9 | 22.9* | 20.9* |
|  | Hypertension+ HF | 14.0 | 14.0 | 7.70 | 19.1 | 12.9 |
|  | Hypertension+ asthma | 5.10 | 3.40 | 3.10 | 5.10 | 4.60 |
|  | Asthma | 5.70 | 4.60 | 1.70 | 3.80 | 3.90 |
|  | Asthma+ RVI | 2.80 | 1.70 | 2.50 | 1.30 | 3.90 |
|  | Hypertension +RVI | 2.40 | 7.70 | 2.90 | 4.30 | 3.30 |
| Current medications | | | | | | |
|  | Oral | 46.2* | 24.7* | 23.5* | 48.8* | 33.4* |
|  | Insulin only | 9.30 | 6.40 | 7.80 | 9.90 | 3.80 |
|  | Oral + Insulin | 5.20 | 5.50 | 3.50 | 9.0 | 7.80 |
| Number of medications used | | | | | | |
|  | < 5 | 49.9* | 29.6* | 24.8* | 57.8 | 35.9 |
|  | > 5 | 10.5 | 7.40 | 9.10 | 9.40 | 7.70 |
| FBS Level | | | | | | |
|  | < 126 mg/dl | 7.40* | 2.30 | 0.60* | 7.10 | 6.30 |
|  | > 126 mg/dl | 53.3 | 35.0 | 33.6 | 60.1 | 37.3 |
| Body mass index | | | | | | |
|  | Normal | 47.2* | 27.6* | 26.1 | 55.1 | 35.8 |
|  | Obesity | 13.4 | 9.70 | 8.0 | 12.2 | 8.0 |
| HbA1c | | | | | | |
|  | < 6.4% | 0.90 | 0.90* | 0.90* | 0.60 | 0.60 |
|  | > 6.4 % | 59.2 | 35.2 | 32.3 | 66.0 | 42.8 |
| Presence of diabetes complication | | | | | | |
|  | No complications | 20.2* | 10.5* | 12.5* | 24.8* | 19.4 |
|  | Complications | 40.3 | 26.8 | 21.7 | 42.5 | 24.5 |

** P value < 0.05, HF=Heart Failure, RVI= Retroviral infection or HIV/AIDS*
